# Nurse-identified patient care and health services research priorities in the United Arab Emirates: a Delphi study

**DOI:** 10.1186/s12913-019-3888-5

**Published:** 2019-01-29

**Authors:** Nabeel Al-Yateem, Muna Al-Tamimi, Maria Brenner, Hanan Al Tawil, Alaa Ahmad, Sharon Brownie, Shameran Slewa-Younan

**Affiliations:** 1 Department of Nursing, College of Health Sciences, University of Sharjah, P.O.B 27272, Sharjah, United Arab Emirates; 2 Research Institute for Medical and Health Sciences (RIMHS), University of Sharjah, Sharjah, United Arab Emirates; 3 School of Nursing, Midwifery and Indigenous Health, Faculty of Science, Charles Sturt University, Orange Campus, Orange, NSW Australia; 4 Trinity College, Dublin, Ireland; 5 Sheikh Khalifa Medical City, Abu Dhabi, United Arab Emirates; 6 School of Nursing and Midwifery, Aga Khan University, Karachi East Africa, Kenya; 7 School of Medicine, Griffith University, Griffith, Australia; 8 Oxford PRAXIS Forum, Green Templeton College, Oxford University, Oxford, UK; 9 Mental Health, Translational Health Research Institute, School of Medicine, Western Sydney University, Sydney, Australia; 10 Centre for Mental Health, Melbourne School of Population and Global Health, University of Melbourne, Melbourne, Australia

**Keywords:** Nursing research, Evidence-based healthcare, Evidence-based practice, Delphi technique, United Arab Emirates

## Abstract

**Background:**

The need for improved research on ill health has been recognized internationally and locally in the United Arab Emirates (UAE). The UAE Nursing and Midwifery Council recently committed to enhancing the status and contributions of nursing in healthcare research across the UAE by establishing a National Committee for Research Development. This study using a Delphi method to identify research priorities from the perspective of nurses delivering frontline healthcare.

**Methods:**

A two-phase Delphi design was implemented with 1032 nurses participating in phase one of the study and 1339 in phase two.

**Results:**

The most important priority was patient safety and healthcare professionals’ awareness of international patient safety goals (including staffing levels and shift length) and potential effects on patient safety. Other important priorities were infection control practices and management of communicable diseases.

**Conclusions:**

These priorities may inform nursing research programs to improve patient care and health outcomes in the UAE and similar contexts worldwide.

## Background

Identifying and setting research priorities for healthcare planning and delivery is central to effective and efficient health services [1–3]. The need for collaborative research among nurse researchers and other health professionals is well documented, and that for improved research on ill health has been recognized internationally [4–7]. These previous reports highlight the need to identify and prioritize research areas for nursing.

Nurses are the largest cohort in the health sector, and are well-positioned to contribute to enhancing standards of care and improving health systems [8, 9]. However, there is a dearth of knowledge about research priorities for the nursing profession in the United Arab Emirates (UAE). Previous studies have addressed research priorities for specific nursing specialties and school nursing [10, 11]; however, no UAE-based studies have identified and ranked research priorities for the general nursing profession.

The UAE is a Middle Eastern nation comprising seven emirates covered by three health authorities: the Ministry of Health and Prevention (MOHaP), the Dubai Health Authority (DHA), and the Department Of Health - Abu Dhabi (DOH). The MOHaP, DHA, and DOH recently published strategic plans for 2017–2021 [12–14] that focus on delivering high quality health services. These strategic plans also cover identified health system priorities, which include non-communicable diseases, cancer, mental health, and respiratory problems. The nursing profession has an important role to play in achieving such aspirations. There are currently 33,429 nurses and midwives employed in various roles across the public and private sectors in UAE. The nursing and midwifery population comprises a range of cultures, predominately Pilipino, Indian, Pakistani, and people from Western and other Arab countries. These nurses and midwives come to the UAE with varying education and skill levels.

**Fig. 1:**
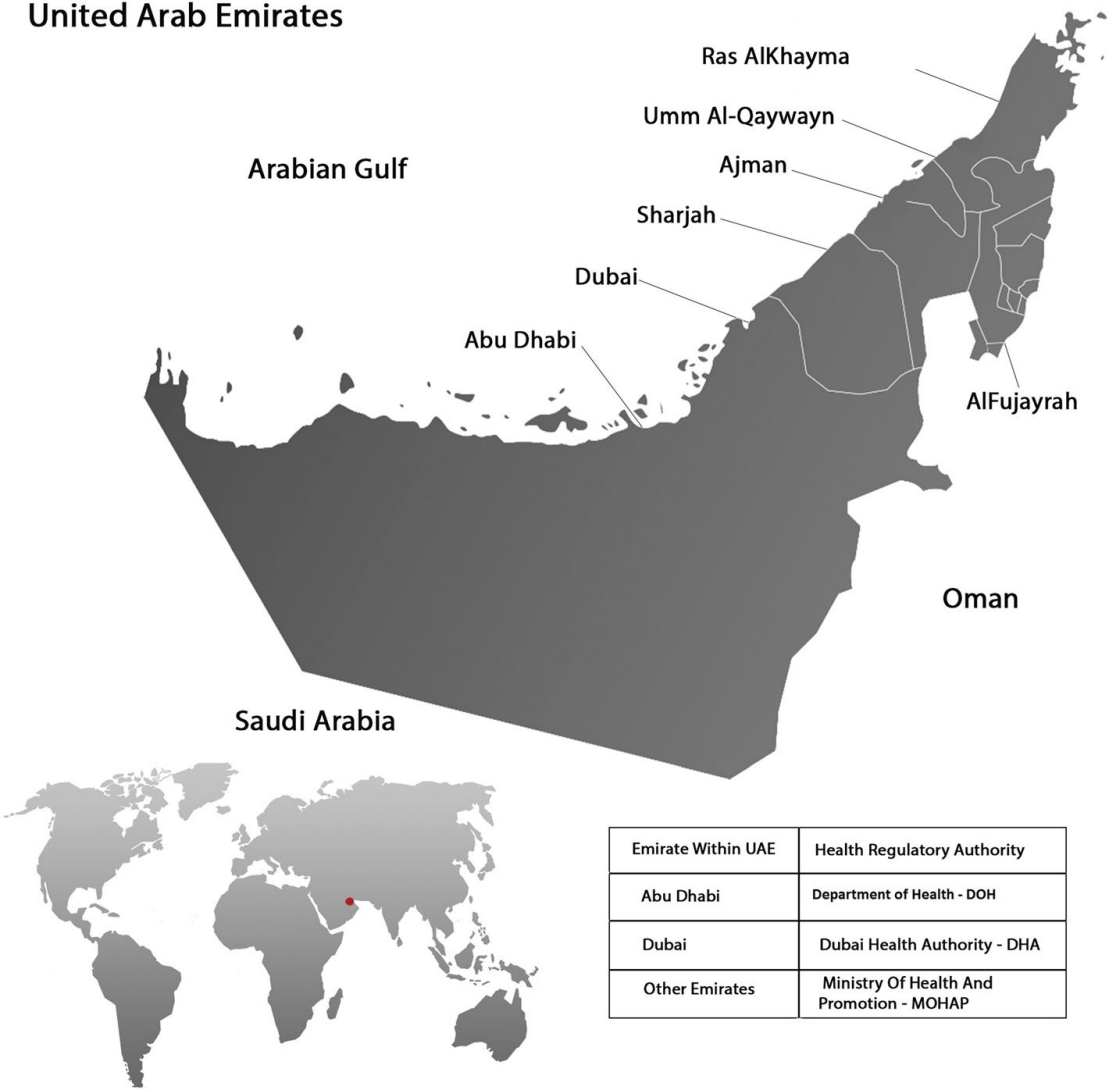
Map of the United Arab Emirates and the health authorities

The UAE Nursing and Midwifery Council recently strengthened the membership and activity of its Research Committee, which represents a significant step in enhancing the status and contributions of nursing across the UAE [15]. The Research Committee is tasked with developing research infrastructure, setting strategic directions, and identifying research priorities to expand practice, education, and leadership. The present study aimed to support these efforts by engaging the nursing workforce to identify and rank research priorities for nurses delivering frontline healthcare. The findings are expected to facilitate a more informed program of research, thereby improving outcomes for patients and their families Fig. 1.

Setting health services research priorities is a concept that emerged in the US, with nursing one of the first healthcare professions to engage in this process [16, 17]. In 1975, Lindeman conducted a landmark US study on clinical practice research priorities [18] in which nurses identified priorities for the discipline of nursing and highlighted their appreciation of the need to explore health service priorities in a wider multi-disciplinary context. Despite this early reference to multi-disciplinary and broader health system contexts, most subsequent studies focused on enhancing nurses’ contributions to health services through encouraging targeted research programs to improve specialty-specific practice outcomes, rather than informing overall health policy [19–21]. For more than 20 years, most studies on nursing research priorities focused on specialist nursing practice, including correctional nursing [1]; care of children [22, 23]; health services, systems, and nursing administration [2]; and school nursing [10, 20]. In addition, many existing studies focused on specific geographic areas such as Australia and New Zealand [24, 25]; Ireland [26, 27]; Europe [23]; the Americas [19]; Africa [28]; and Eastern Mediterranean countries [29].

Many previous studies identified nursing research priorities using consultative processes that included: key medical, health policy, academic, and allied health professionals; members of professional and statutory nursing organizations; funding agencies; and nurses in practice, management, education, and research roles. Some studies identified broad priorities, but offered little direction for developing scientifically verifiable standards to improve nursing practice and overall healthcare delivery. However, recent studies have focused on nurses and midwives identifying priorities for their own professions/clinical specialties to improve clinical practice [1, 22, 23, 30, 31]. Health research priorities are highly context-dependent. They vary according to factors such as: geographic location or country; prevalent culture; and the nature, status, and focus of the healthcare system. Variations in these factors mean that it is necessary to replicate studies on identifying research priorities in different countries. Consistent with this trend, the present study aimed to give voice to nurses across the entire UAE health system and capture their views about/ranking of nursing and broader health service research priorities (All-party Parliamentary Group on Global Health, 2016; Deloitte, 2017).

## Methods

### Design: The Delphi method

The Delphi method was first developed to examine the impact of technology on warfare in 1950s, and has been defined as a type of consensus method (often using non-face-to-face techniques) for structuring a group communication process and allowing a group of individuals to deal with a complex problem [32]. It has been proven to be a feasible method when developing culturally-adapted interventions in mental health for minority and diverse groups [33]. The Delphi technique involves distribution of online questionnaires to a group of experts, with responses being anonymous. This method involves a number of iterations before consensus is reached. Feedback from the expert group as a whole is obtained to assist panelists to assess their ratings against the group feedback. This process involves eight steps: 1) framing a research question, 2) formation of the panel, 3) determining the expert panel size, 4) constructing the questionnaire, 5) providing information to panel members to aid their judgments, 6) administering the questionnaire, 7) analyzing response rounds and providing feedback to the panel, and 8) reporting results.

### Setting

This study was conducted across private- and public-sector hospitals in the UAE.

### Sample

Sample size calculations for the present study indicated that at least 1000 nurses were needed to achieve the desired power level. The calculations were based on an estimated population size of 33,429 nurses working across the UAE, a 95% confidence level, and a confidence interval of 3 [34]. The inclusion criterion was staff nurses who had worked in clinical settings in the UAE healthcare system for more than 1 year. Nurses with less than 1 year of experience were excluded as they might not have had sufficient exposure to the healthcare system to identify relevant research priorities.

A multistage clustered sampling procedure was initially considered appropriate to achieve the goals of the study. However, given difficulties in obtaining information about all UAE hospitals and resistance from many hospitals to recruitment of participants, the sampling procedure was changed to non-probability sampling. Therefore, nurses who met the inclusion criterion in all known hospitals that were accessible (i.e., gave permission to conduct the study) to the research team were invited to participate in the study. This included private and government-run hospitals, and hospitals managed by independent governing bodies (e.g., the DHA and DOH- Abu Dhabi) from across the UAE.

### Data collection

Two questionnaires were used to capture data for the two rounds of this study. The round one questionnaire included an open-ended question that invited participants to identify five research priorities that they considered most important to enhance the general delivery of nursing care in their hospital. Participants’ demographic data were also gathered, including age, clinical setting, clinical grade, emirate in which they worked, qualification level, and years of experience.

The questionnaire for round two was developed from responses received in round one. Identified research priorities were placed randomly in the questionnaire, and round two participants were asked to rate the importance of each research priority on a 7-point Likert scale (1 = low importance to 7 = high importance). Demographic data were not collected in round two to decrease repetition and survey burden on participants, and improve the response rate. However, clinical setting and emirate were extracted from returned questionnaires. Typically, round two participants would be a subset of those from round one, but the variable length of hospital administrative procedures necessary to gain permission for data collection meant that more hospitals were able to participate at the time round two started. The addition of these hospitals meant that demographic information was not available for some round two participants. However, it was anticipated that demographic data collected in round one would provide adequate general information about the study population.

Hospitals that approved participation in the study were consulted about the survey medium (electronic or paper-based) that best suited their organization and staff. Those that selected electronic surveys were asked to provide a list of email addresses for their nursing staff. An invitation letter containing information about the study and a link to an online questionnaire was then emailed to participants. Hospitals that selected paper-based surveys were asked to provide information about their departments and number of staff in each department. Those hospitals were provided with hard copies of the invitation letter and questionnaire, which were distributed and collected centrally through their education or research offices. The same data collection process was followed in both rounds of the study.

### Data analysis

Responses to the open-ended question in round one were analyzed qualitatively, assisted by qualitative data analysis software (Atlas.ti V7®). Participants’ statements about research priorities were read and key clinical research areas reported were highlighted as codes. Similar codes were grouped together and categorized into broad issues, which were considered initial research priorities (i.e., patient safety and awareness of international patient safety goals, including nursing staffing issues and shift length). Initial research priorities were then used to construct the second questionnaire (round two), with each priority represented as an item in the constructed questionnaire. Analysis of round two results entailed examination of the rating scores for each initial research priority as ranked on a 7-point Likert scale.

Because healthcare research activity in the UAE, especially among nurses, is still in its infancy [35], the study team agreed to select the highest-ranked research priorities to focus on as a starting point. Therefore, in regard to the consensus cut-off points, the group agreed that the criterion used to determine consensus that a research priority was of high importance was that the identified priority was rated as important/very important by at least 90% of participants. Priorities that were rated as important/very important by at least 75% of participants were considered of medium importance. The remaining priorities were considered of low importance.

## Results

### Participants

In total, 1032 participants completed and returned the round one questionnaire, representing a 56% response rate (1830 questionnaires distributed). Participants were from across the emirates including Sharjah, Ajman, Fujairah, Dubai, and Abu Dhabi. The majority of participants were staff nurses, female, had a diploma-level qualification, and 5–10 years of experience. There were 1339 participants in round two, representing a response rate of 66% (2000 questionnaires distributed); these participants were from various clinical settings. Participants’ demographic details are presented in Table 1.

**Table 1:** Demographic data for round one and round two participants

		*n* (%)
Round one (*n* = 1032)		
Gender	Female	838 (81.2)
	Male	127 (12.3)
	Missing	67 (6.4)
Emirate	Ajman	83 (8.0)
	Sharjah	468 (45.3)
	Dubai	236 (22.9)
	Abu Dhabi	245 (23.7)
	Fujairah	0 (0.0)
Clinical Setting	General nursing	241 (23.4)
	Pediatric ward or setting	182 (17.6)
	Emergency department	142 (13.8)
	Operating theatre	125 (12.1)
	Maternity and labor ward	134 (13.0)
	Adult critical care units	169 (16.4)
	Long-term care facility (geriatric)	31 (3.0)
	No answer	8 (0.7)
Grade	Staff nurse	672 (65.1)
	Charge nurse	209 (20.3)
	Nurse educator	54 (5.2)
	Nurse administrator	22 (2.1)
	Other	17 (1.6)
	No answer	58 (5.6)
Qualification	Diploma	514 (49.8)
	Bachelor’s	421 (40.8)
	Post graduate diploma	45 (4.4)
	Master’s	43 (4.2)
	No answer	9 (0.8)
Experience	1–2 years	124 (12.1)
	2–5 years	158 (15.3)
	5–10 years	330 (32.0)
	More than 10 years	323 (31.3)
	No answer	97 (9.4)
Round two (*n* = 1339)		
Emirate	Ajman	303 (22.6)
	Sharjah	415 (31.0)
	Dubai	277 (20.7)
	Abu Dhabi	202 (15)
	Fujairah	142 (10.6)
Clinical Setting	General nursing	223 (16.7)
	Pediatric ward or setting	267 (19.9)
	Emergency department	175 (13.1)
	Operating theatre	160 (11.9)
	Maternity and labor ward	176 (13.1)
	Adult critical care units	308 (23.0)
	Long-term care facility (geriatric)	30 (2.2)

### Research priorities

Round one identified 31 general nursing research priorities, which were rated in terms of importance by round two participants. Based on the established criteria, five research priorities were identified as highly important and 26 were considered of medium importance. No priorities were considered of low importance. The most important research priority was patient safety and healthcare professionals’ awareness of international patient safety goals. This included nursing staffing issues such as shift length and the flexibility of the work schedule and the potential effects on patient safety, and was rated as important by 91.5% of participants. The second most important research priority was infection control practices in different hospital departments, which was rated as important by 91.2% of participants. The third most important research priority was the interpersonal skills of nursing staff, including critical thinking, communication skills, problem solving, time management, and planning skills and the effect of such skills on care outcomes. This was rated as important by 90.4% of participating nurses. The fourth research priority was medication management in clinical settings, which was rated as important by 90.1% of participants. Finally, 90% of participants considered pain assessment and management policies and practices in the UAE to be important. Table 2 presents research priorities of high and medium importance for the nursing profession in the UAE, as ranked by participating nurses.

**Table 2:** Research priorities of high and medium importance for the nursing profession in the United Arab Emirates

		Importance rating	
Highly important research priorities		n	%
1	Patient safety and awareness of international patient safety goals including nursing staffing issues and shift length	1225	91.5
2	Infection control practices and management of communicable diseases in different hospital departments	1221	91.2
3	Critical thinking, communication skills, problem solving, time management, and planning skills for nurses	1210	90.4
4	Issues related to medication management in clinical settings	1206	90.1
4	Pain assessment and management	1205	90.0
Medium important research priorities			
1	Implementing up-to-date, evidence based nursing care and care pathways	1190	88.90
2	Improving communication and team work between healthcare professionals	1190	88.90
3	Work environment, work conditions, staff satisfaction, stress levels, available resources and safety for nurses	1187	88.60
4	Nurses role in prevention of and caring for patients with non-communicable diseases (e.g., cardiac, diabetes, asthma, cancer)	1187	88.60
5	Continuous education and professional development for nurses to maintain competency	1190	88.30
6	Care of immobilized, unconscious, and bedridden patients and prevention of bedsores and ulcers	1182	88.30
7	Wound care management	1180	88.10
8	Leadership and management styles and skills of nurse managers	1177	87.90
9	Health teaching related to current illnesses in different specialties setting and after discharge	1176	87.80
10	Evaluating outcomes and quality of nursing care, and factors affecting it	1176	87.80
11	Strategies to manage staffing issues and shortage in staff	1170	87.40
12	Health education and promotion role of nurses as related to the wider community	1164	86.90
13	Use of technology in nursing care: benefits and drawbacks	1161	86.70
14	Continuous measurement of patient satisfaction of nursing care in different clinical settings	1141	85.20
15	Legal and ethical issues related to the role of nurse	1141	85.20
16	Nurse patient relationship, dealing with different type of patients and violence against nurses in the work place	1139	85.10
17	Antibiotic use and prescription	1139	85.10
18	Special care needs of vulnerable and high risk populations	1134	84.70
19	The need for advanced and specialized nursing care	1127	84.20
20	Ensuring suitable clinical environment for patients from different age groups including family centered care	1126	84.10
21	Learning local culture and language to provide comprehensive care	1120	83.60
22	Rehabilitation, palliative, and end of life care	1104	82.40
23	Care of patients with cancer	1101	82.20
24	Attending for psychosocial needs of patients while being admitted for treatment or surgery in hospitals	1084	81.00
25	Assisting graduate nurses and new recruits transitioning into their nursing career	1060	79.20
26	Utilization of care assistants, and none professional support staff to improve patients care	1053	78.60

## Discussion

The Delphi methodology used in this study was robust, as the goal was reaching consensus among study participants on priority research areas for the nursing profession in the UAE. Delphi studies are used extensively worldwide, and the literature indicates that this design is the most appropriate for studies of this nature [1, 2, 23]. Delphi studies vary in terms of sample size and type of participants. In similar studies identifying research priorities for nursing staff, the sample size ranged from 40 to 1695, and included staff nurses, academics, and nurse clinicians [1, 2, 19, 27, 28]. Therefore, the large sample size in the present study and inclusion of nursing staff from different clinical settings, specialties, levels, and from across the UAE is congruent with similar published studies. This strengthened the results, enhanced generalizability, and ensured the clinical relevance of the present study.

The outcomes of the present study may contribute to enhancing research-based practice and developing the nursing profession in the UAE and internationally. Research-based practice is essential in developing the profession’s theoretical base and to generate evidence underpinning nursing practice. Through engaging in research, nurses contribute to improving the health of individuals, families, communities, and populations. However, maximum benefits can be achieved when research efforts are focused and prioritized. Hinshaw (1997) examined the benefits of research priorities projects worldwide, and found an exponential growth in research after research priorities by the US National Institute of Nursing Research were identified through the National Nursing Research Agenda Project [17].

The findings of the present study are important in supporting nurses in gaining funding for research projects. Gaining research funding is highly competitive; the UAE is no exception, and this study may support nurses’ funding applications in high-need research areas [16]. Furthermore, the transition of nursing education to higher education institutions mandated nurse academics to produce high-quality research similar to that of their peers, and caused a separation between nursing research and the clinical setting. This makes it necessary to build nurses’ capacity for research (clinicians, educators, nurse managers, and leaders) and set clinically-informed research priorities to guide the nursing research agenda across the UAE [36].

Nursing is a major component of the healthcare system, and has an important part to play in ongoing healthcare service development. It is noteworthy that three of the five highly important priorities identified in this study focused on patient safety (and awareness of international patient safety goals), medication, and pain management for patients in different clinical areas in the UAE. Similar findings were identified in a study in Sweden, where identified priorities aimed to improve clinical practice, assure patients’ wellbeing, and create a caring environment [37]. Other key priority areas identified in this study were the work environment for nurses and interpersonal skills (e.g., critical thinking, communication skills, problem solving, time management, and planning skills). Participants identified these issues as factors that can affect patient care outcomes. This is consistent with a previous study in Ireland that identified staffing issues in practice, communication in clinical practice, and recruitment and retention of nurses as highly important research areas [27].

A final point worth considering is whether the research priorities identified in the present study and similar international studies reflect actual research needs, or gaps in nurses’ knowledge in these areas. This concern was raised in a study by Brenner et al. (2014) that sought to identify research priorities for pediatric nursing in Ireland. In that paper, the authors indicated that some identified priorities (specifically those related to pain management and assessment) might reflect a gap between nurses’ knowledge and available evidence or suboptimal application of evidence in clinical practice, as evidence in that area is well advanced [22].

Finally, there are several limitations within this study, the nurses that participated in this study were predominantly diploma-qualified, which reflects the demographics of the nursing workforce in UAE. Their education and competency in understanding the research process (which might be limited by their educational preparation), might have affected their ability to identify accurate research priorities. In addition, it is worth noting that the reported gaps in nurses’ knowledge of currently available clinical evidence could have impacted participants’ suggested research areas, which may in fact reflect educational need rather than actual research priorities.

## Conclusions

This study used Delphi methodology to identify and rank research priorities for the nursing profession in the UAE. These identified priorities should be used to guide a more informed research program with the overarching aim of improving outcomes for patients and their families. These priorities may also support nurse researchers in conducting relevant research projects, and enable nurses to contribute their greatest therapeutic value. This study suggests that nurse researchers in the UAE should focus future projects on these identified research priorities. The findings from this study may also directly inform nurse-led research in similar contexts, as well as similar studies investigating nurse-identified research priorities in other parts of the world.

### Implications of study results

- The priority areas identified in this study are ideally situated to guide a relevant and effective national nursing research agenda in the UAE.
- Researchers in the fields of nursing and healthcare in UAE may consult the list of identified research priorities when conducting their research studies. This will ensure that the research they conduct is aligned with current, clinically relevant issues and priorities identified by their colleagues in clinical settings.
- Health service managers and policy makers may also consult this list of research priorities when prioritizing and allocating funds for health and nursing research in the UAE.

## Abbreviations

DHA: Dubai Health Authority
DOH: Department of Health- Abu Dhabi
MOHaP: Ministry of Health and Prevention
UAE: United Arab Emirates
